# Dolphins Adjust Species-Specific Frequency Parameters to Compensate for Increasing Background Noise

**DOI:** 10.1371/journal.pone.0121711

**Published:** 2015-04-08

**Authors:** Elena Papale, Marco Gamba, Monica Perez-Gil, Vidal Martel Martin, Cristina Giacoma

**Affiliations:** 1 University of Torino, Life Sciences and Systems Biology Department, Torino, Italy; 2 Society for the Study of Cetaceans in the Canary Archipelago (SECAC), Lanzarote, Spain; Lund University, SWEDEN

## Abstract

An increase in ocean noise levels could interfere with acoustic communication of marine mammals. In this study we explored the effects of anthropogenic and natural noise on the acoustic properties of a dolphin communication signal, the whistle. A towed array with four elements was used to record environmental background noise and whistles of short-beaked common-, Atlantic spotted- and striped-dolphins in the Canaries archipelago. Four frequency parameters were measured from each whistle, while Sound Pressure Levels (SPL) of the background noise were measured at the central frequencies of seven one-third octave bands, from 5 to 20 kHz. Results show that dolphins increase the whistles’ frequency parameters with lower variability in the presence of anthropogenic noise, and increase the end frequency of their whistles when confronted with increasing natural noise. This study provides the first evidence that the synergy among SPLs has a role in shaping the whistles' structure of these three species, with respect to both natural and anthropogenic noise.

## Introduction

Environmental background noise is highly variable, in both time and location [1]. Many sources of biotic and abiotic origins contribute to ambient noise in the ocean, such as wind and waves, precipitation, seismic processes, thermal events, biological and anthropogenic activities. The anthropogenic component is especially significant in areas involved in offshore activities, along commercial shipping routes, in zones of intense fishing and in developed coastal areas. As ocean noise levels increase, there is increasing concern for the conservation and management of marine mammal species, which strongly rely on acoustics for orienting, hunting and communicating [2, 3].

Cetaceans' auditory systems have evolved to deal with small fluctuations in natural noise levels [4], so that larger fluctuations in anthropogenic noise have a potential to cause a number of negative consequences, spanning from the disruption of interactions to decreased hunting efficiency [5]. The potential impact of spatial, spectral and temporal persistence of noise may be either chronic or short term, and differs among species [6], with the activity state of animals [7, 8], or the acoustic habitat [9].

High noise levels, spanning across several frequency bands, can mask relevant signals [1, 10, 9, 3, 11, 12]. Individuals may cope with background noise by enacting compensation mechanisms, such as changing a signal’s amplitude, duration, repetition rate and/or frequency [13]. This ability to overcome masking noise by altering vocalizations may be crucial for communicating in a dynamic acoustical environment [3]. Nevertheless, whether and to what extent marine animals are able to compensate for environmental noise remains unclear. Jensen et al. [14] have suggested that changes in acoustic behaviour could be costly, because of possible increases in energy expenditure, modifications of the original information content, or increased risks of detection by predators.

For small odontocetes, an effect of noise on the acoustic behaviour and on the structure of signals was hypothesized and identified in a frequency shift of tonal sounds. Morisaka et al. [15] suggested a use of lower frequencies in presence of stronger environmental noise (not attributable to anthropogenic origin, [1]) in Indo-Pacific bottlenose dolphins, while May-Collado et al. [16] suggested that signals emitted by bottlenose dolphins may have a higher maximum, end frequency and range (maximum—minimum frequency) in Atlantic areas where strong low-frequency noise is generated by dolphin-watching boats. Marine traffic was also hypothesized to cause temporary shifts in maximum, minimum and beginning frequency in a resident population of bottlenose dolphins occurring in the Sado estuary in Portugal [17]. In the Mediterranean Sea, La Manna et al. [18] found that a longer signal duration and higher maximum frequency were used by bottlenose dolphins in presence of anthropogenic noise, while Azzolin et al. [19] found shorter duration and higher end frequency in signals emitted by striped dolphins in a naturally noisy environment. All the above evidence confirms that noise has an impact on the communication systems of dolphins, but this depends on various factors, such as the species involved and noise’s characteristics. This study intends to evaluate whether three species of dolphins respond consistently to a continuous background noise.

In this this study we tried: i) to quantify the noise levels to which species are exposed in the area of the Eastern Canary Islands, a strategic crossroads along the major shipping lanes of the Atlantic Ocean, and ii) to assess the basic acoustic structure of simultaneously collected dolphin whistles. In other words, we aimed at investigating the temporary, short-term consequences of noise levels on the acoustic structure of dolphin’s whistles. Since frequency parameters are the most stable within dolphin populations [20, 21, 22, 23, 19, 24,25] and scarcely linked to social and behavioural factors, we analysed changes in these parameters occurring in three dolphin species (*Delphinus delphis, Stenella coeruleoalba* and *Stenella frontalis*).

We predicted that dolphins enact different strategies to compensate for natural ambient noise, depending on the species-specific features of their whistles and local characteristics of noise.

Two other alternative scenarios can be predicted: (1) no compensation occurs and consequently we will find no differences in whistles frequency parameters emitted under lower and higher noise; (2) different species enact a common strategy, giving rise to convergent shifts in frequency parameters.

## Material and Methods

### Statement of ethics

The *Dirección General del Medio Natural, Consejería de Medio Ambiente y Ordenación Territorial, Gobierno de Canarias* authorized data collection (under Decree 20/2004, 2^nd^ March: approval of the *Reglamento Orgánico de la Consejería de Medio Ambiente y Ordenación Territorial*- which attributes to the *Dirección General del Medio Natural* the function of authorizing for the observations of cetaceans having scientific, education, technical, cultural or conservation aims, into the article 31. 17), under:

- Decree 178/2000, 6^th^ September, for the regulation of the activities of cetaceans observation;
- Decree 1727/2007, 21^st^ December, for the establishment of measures for cetaceans protection;
- Law 42/2007, 13^th^ December, Natural Heritage and Biodiversity.

### Data collection

**Fig 1:**
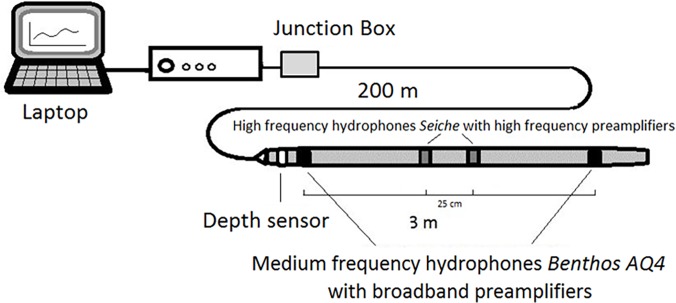
Scheme of the hydrophone system and its geometry. The towed linear array is composed by 2 Medium frequency hydrophones *Benthos* AQ4 (Benthos, Falmouth, USA) with broadband preamplifiers HP/02 (Magrec, Devon, UK) incorporating a low cut filter set to provide -3dB at 100Hz, and 2 spherical ceramic hydrophone elements (Seiche UK Ltd) with high frequency preamplifiers with a 2kHz low cut filter. A 200m cable connected the array with the electronic component through a junction box. Recordings were digitalized at a sampling rate of 192 kHz and directly visualized on a laptop.

This study was carried out between 2008 and 2012 in the waters surrounding the Eastern Islands of the Canary archipelago (Lanzarote, Fuerteventura and Gran Canaria) where the three dolphin species (short-beaked common dolphin (*Delphinus delphis*), striped dolphin (*Stenella coeruleoalba*) and Atlantic spotted dolphin (*Stenella frontalis*) regularly occur. Recordings were collected in open and deep waters, using a towed linear array with 4 elements (Fig. 1): including 2 Medium Frequency hydrophones (Benthos AQ4: Benthos, Falmouth, USA) spaced 3 m apart (voltage sensitivity of -201 dBv ref. 1 μPa ± 1 dB @ 20°C and frequency response of 1 Hz-15 kHz (±1.5 dB)). Hydrophones were fed by a matched pair of broadband preamplifiers HP/02 (Magrec, Devon, UK) incorporating a low cut filter set to provide -3 dB at 100 Hz; and 2 spherical ceramic hydrophone elements having a frequency response of 2–150 kHz (Seiche UK Ltd) potted with high frequency preamplifiers with a 2 kHz low cut filter (sensitivity of the front element was -161 dB re 1 V/μPa and the rear element was -158 dB re 1 V/μPa). Recordings were digitalized at a sampling rate of 192 kHz. To minimise the effect of noise emitted by the research boat, the engine was maintained in neutral for most of the time (dB re 1 μ Pa band 4.4–5.6 kHz: 86.89; dB re 1 μ Pa band 5.6–7.1 kHz: 80.25; dB re 1 μ Pa band 7.1–8.9 kHz: 79.21; dB re 1 μ Pa band 8.9–11.2 kHz: 81.96; dB re 1 μ Pa band 11.2–14.1 kHz: 77.39; dB re 1 μ Pa band 14.1–17.8 kHz: 81.45; dB re 1 μ Pa band 17.8–22.4 kHz: 79.83). To prevent considering signals from other groups or species out of the visual range, we did not analyse recordings when visibility was less than 500 m, or when more than one species was sighted, or when low amplitude whistles were difficult to discern from the background noise. Data collection was carried out between 08:00 and 20:00, during all the inherent activities of the animals, when wind intensity was lower than 4 on the Beaufort scale and sea state lower than 4 on the Douglas scale (following Evans et al. [26], condition 3 is the weather state over which the probability to individuate cetaceans on the sea surface is limited and, consequently, surveys are carried out usually within this condition). We collected a total of 104 recordings (each corresponding to a different sighting): 84 for Atlantic spotted dolphin consisting of 12.08 hours, 26 for short-beaked common (3.23 hours) and 16 for striped dolphin (3.15 hours).

### Acoustic analyses

Since the aim of our study was to describe signal variation in relation to noise, we extracted from each recording, all the sounds showing a strong signal-to-noise ratio, and that did not overlap with other vocalisations. Furthermore, we identified the presence of a whistle by analysing Sound Pressure Levels (SPLs) in the one third octave bands of the signals. We considered the presence of a signal when the SPLs were higher than at least 20 dB over the background noise.

**Fig 2:**
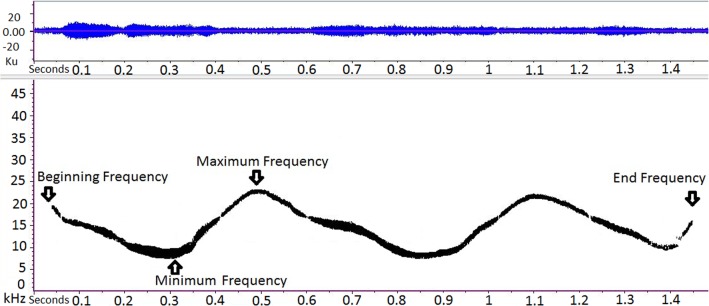
Spectrogram of a dolphin's whistle. Parameters considered in the study are pointed out. All the sounds showing both a strong signal to noise ratio and not overlap with other vocalisations were extracted. The presence of a whistle was also identified by analyzing the SPLs in the one third octave bands of the signals: we considered the signals when the SPLs were higher of at least 20 dB. We measured four signal parameters for each whistle: beginning frequency, end frequency, minimum frequency and maximum frequency.

For each whistle we measured four frequency parameters i.e. beginning, end, minimum and maximum frequencies (Fig. 2). Other acoustic parameters, such as temporal parameters and signal modulation, were not taken into account because they could be influenced by social and behavioural factors [16], which we could not control in our sampling conditions.

Frequency parameters were measured using the spectrogram (time versus frequency graph) view in CoolEdit 2000 (Syntrillium Software, U.S.A.) with spectrogram resolutions of 256–512 band, 256 FFT size and Hamming window, and were verified with the semi-automatic software for acoustic analysis included in MATLAB (the MathWorks Inc., U.S.A.). Whistles that presented similar contours were measured only once, because a sequence of stereotyped whistles could result from behavioural and/or social interactions and would increase pseudoreplication [27, 28, 29]. We considered each recording as an independent sample and used for the analysis the mean value of each parameter of the whistles within the recording.

In order to compare our results with prior studies, we approximated noise analysis to banks of one-third-octave filters for communication frequencies (following ANSI standard S1.6-1984, see also [1]). Since the mean values of signal parameters ranged from 5.2 to 23.1 kHz, we included 7 one-third-octave bands with central frequencies from 5 to 20 kHz ranging from 4.4 to 22.3 kHz. Environmental background noise was measured as SPLs in dB re 1 μ Pa using PRAAT [30]. We used a custom PRAAT script to extract SPLs from the central frequencies of each one-third-octave band. All values were saved to a tab-delimited text file and exported to Microsoft Excel [31].

To obtain only one set of noise values for each sighting, background noise estimation was performed on recordings of maximum 10 sec, throughout the period before or after a sequence of whistles. It was not possible to extract our vessel flow noise from the ambient environment measurements, but since the boat’s engine was essentially maintained in neutral during all samplings, we expected that the environmental background noise was by far the dominant noise. We classified standard survey weather conditions along a scale ranging from 0 to 3. We evaluated the mean level of environmental background noise in good weather conditions (0–1) (A—low noise), and in bad weather conditions (2–3) (B—medium noise) for which we were sure that no boats were present within one mile from the research vessel, in the range comprised between 4.4 kHz and 22.3 kHz (Table 1). We pooled in a third category (C—high noise) all recordings with SPLs higher than those collected in the worst weather conditions, by assuming that such high levels in mid-frequency bands could be attributed to the cavitating propellers of passing vessels, which are audible over long distances [1, 32].

**Table 1:** Sound Pressure Levels of environmental background noise. The noise considered “in good weather conditions” was measured as the mean levels in conditions 0–1 (category A); the one “in worst weather conditions” as the mean value of the recordings collected in the conditions 2–3 (category B), for which no boats were present within one mile from the research vessel. The last typology (category C) represents the mean level of the noise overcoming the one recorded in the standard weather conditions (following Evans et al. [26] we considered standard weather conditions for carrying out sea surveys, the level of the sea on the Douglas scale and of the wind on the Beaufort scale from 0 to 3).

	dB re 1 μ Pa band 4.4–5.6 kHz	dB re 1 μ Pa band 5.6–7.1 kHz	dB re 1 μ Pa band 7.1–8.9 kHz	dB re 1 μ Pa band 8.9–11.2 kHz	dB re 1 μ Pa band 11.2–14.1 kHz	dB re 1 μ Pa band 14.1–17.8 kHz	dB re 1 μ Pa band 17.8–22.4 kHz
A							
Background noise in weather conditions (0–1)	31.69	32.57	32.71	34.57	39.32	47.66	41.81
Standard deviation	11.26	10.14	11.68	12.37	8.31	17.50	8.81
B							
Background noise in weather conditions (2–3)	75.45	73.14	72.55	69.07	67.02	66.65	66.54
Standard deviation	10.45	12.41	10.71	11.41	10.71	10.08	8.42
C							
Background noise over A and B conditions	109.92	108.35	108.82	110.90	101.17	103.08	96.85
Standard deviation	2.59	2.97	5.66	6.74	5.43	6.69	5.75

### Statistical analysis

The statistical software package IBM SPSS Statistics 20.0 (IBM Corporation) was used to perform descriptive analyses of the dataset. To describe the degree of variability of frequency parameters, we calculated the Coefficient of Variation (CV) ((standard deviation/mean)*100). We evaluated the normality in distribution of whistle frequency and SPL data by Kolmogorov–Smirnov test.

We tested for differences among noise levels observed during the recordings of each species, by one-way ANOVAs, as well as for differences among the mean values of noise levels evaluated in medium noise conditions and the mean levels observed during recordings of each species by Student’s T test.

To evaluate relations between whistle parameters and environmental background noise, for each species we performed 28 correlation tests (four parameters, seven bands). In each correlation test and for each recording we considered the SPL values (dB) in each one-third-octave noise band and the values of the Δ frequency of each parameter. The Δ frequency was calculated by subtracting the central frequency of each one-third-octave band from the mean value of the frequency parameter of the whistles of each recording. Therefore, for correlations we did not use the whistles’ frequencies, but seven differences per whistle among signal value and the central frequency of each band.

Furthermore, linear regressions were then used to test for patterns of variation in whistle frequencies, with respect to the SPLs of each one-third-octave.

Finally, we used linear mixed models to analyse the relationship between whistle frequencies and the SPLs of the two bands contiguous to the noise band overlapping with the mean values of the frequency parameter considered, by taking into account the year of recording as random factor. In this case also interactions among the SPLs of the two bands were tested to evaluate the range of noise influencing frequency parameters.

## Results

### 1. Sound Pressure Levels (SPLs) of environmental background noise

**Fig 3:**
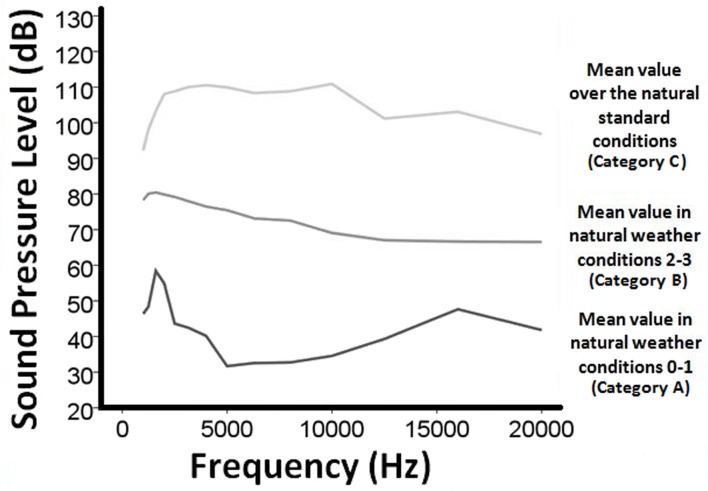
Sound Pressure Levels in dB (ref. 1μPa ± 1dB) in the communication frequency bands from 1 to 22 kHz. We considered for each sighting a recording, of maximum 10 sec, collected throughout the period before or after a sequence of whistles. Sound Pressure Levels (SPLs) of the central frequencies of each one-third-octave band were extracted using PRAAT [30]. The mean level of environmental background noise was analyzed in good weather conditions (0–1) (category A) and in the worst weather conditions (2–3) (category B) for which we were sure that no boats were present within one mile from the research vessel. We pooled in a third category all recordings with SPLs overcoming the ones collected in the worst weather conditions, assuming that the highest levels in mid-frequency bands could be attributed to cavitating propellers of passing vessels, audible over long distances (category C).

The environmental background noise recorded during dolphin sightings did not present impulsive events. SPLs measured in the 4.4 kHz—22.4 kHz range varied from a mean of 31.69 dB re 1 μ Pa in low noise for band 4.4–5.6 kHz to a mean of 110.90 dB re 1 μ Pa in high noise for band 8.9–11.2 kHz (Table 1). SPLs mean values of all 4.4 kHz—22.4 kHz bands of environmental noise recorded during standard survey conditions 0–1 (low noise category A) were lower than values obtained in conditions 2–3 (medium noise category B), and these last ones were lower than data obtained when additional sources of anthropogenic noise were present (high noise category C) (Fig. 3).

Mean values of environmental background noise levels sorted for the species recorded are shown in Table 2. Noise data collected during recordings of Atlantic spotted dolphins showed the lowest SPLs and the highest variability, while the lowest variability was recorded for common dolphins (Coefficients of Variation: 23.3–25.5% for Atlantic spotted dolphin; 20.5–24.7% for striped dolphin; 16.6–19.9% for short-beaked common dolphin). Nevertheless, we found no significant differences among the SPLs observed during recordings of any species (Table 2). Mean SPLs of noise recorded in the presence of each species were always higher than the mean SPLs calculated for category A (Fig. 3) of at least 26 dB and always lower than the mean SPLs calculated for category C. Compared to the mean SPLs for category B, instead, they were significantly higher in the bands 8.9–11.2 kHz, 11.2–14.1 kHz, 14.1–17.8 kHz and 17.8–22.4 kHz except for the striped dolphins for which they were significantly different only in bands 14.1–17.8 kHz and 17.8–22.4 kHz (Table 3).

**Table 2:** Mean, maximum, minimum and standard deviation values of the SPL (dB re 1 μ Pa) measured in 1/3 octave bands in the presence of short-beaked common, Atlantic spotted and striped dolphins’ recordings. Also, results of the one-way ANOVAs between noise levels (in SPLs) for species are shown. No significant differences were found among the SPLs examined.

		dB re 1 μ Pa band 4.4–5.6 kHz	dB re 1 μ Pa band 5.6–7.1 kHz	dB re 1 μ Pa band 7.1–8.9 kHz	dB re 1 μ Pa band 8.9–11.2 kHz	dB re 1 μ Pa band 11.2–14.1 kHz	dB re 1 μ Pa band 14.1–17.8 kHz	dB re 1 μ Pa band 17.8–22.4 kHz
Short-beaked common dolphin **N = 26**	Mean	**78.18**	**77.26**	**77.82**	**78.66**	**74.51**	**78.46**	**76.49**
	Maximum	100.29	102.83	106.51	109.69	94.37	100.8	99.51
	Minimum	52.22	49.36	48.63	54.87	48.50	59.55	49.31
	Sd	14.36	15.09	15.41	15.71	12.39	13.57	13.88
	CV	**18.37**	**19.53**	**19.80**	**19.97**	**16.63**	**17.30**	**18.15**
Atlantic Spotted dolphin **N = 84**	Mean	**76.96**	**76.00**	**75.73**	**75.42**	**71.90**	**74.34**	**72.14**
	Maximum	112.90	109.50	112.28	112.53	107.30	110.86	103
	Minimum	20.30	23.26	19.67	20.77	28.30	30.41	29.74
	Sd	18.1	18.2	18.5	19.2	16.9	17.4	17.7
	CV	**23.58**	**23.97**	**24.43**	**25.48**	**23.56**	**23.37**	**24.53**
Striped dolphin **N = 16**	Mean	**80.47**	**80.42**	**78.95**	**79.05**	**74.87**	**76.16**	**75.21**
	Maximum	111.17	119.79	114.86	119.63	103.21	104.94	104.93
	Minimum	47.51	46.52	50.64	53.12	54.96	54.64	53.94
	Sd	17.67	19.91	18.12	19.57	15.84	16.25	15.45
	CV	**21.96**	**24.76**	**22.94**	**24.76**	**21.16**	**21.33**	**20.54**
**One-way ANOVA F**		0.29	0.43	0.30	0.46	0.42	0.63	0.46
***P value***		0.75	0.66	0.74	0.63	0.66	0.53	0.46

**Table 3:** Statistics of the t-test between the mean SPLs in 2–3 weather conditions (category B) and the mean values recorded in presence of each species. Results were significantly different in the bands 8.9–11.2 kHz, 11.2–14.1 kHz, 14.1–17.8 kHz and 17.8–22.4 kHz for short-beaked common dolphins and Atlantic spotted dolphins. For the striped dolphins they were significantly different in bands 14.1–17.8 kHz and 17.8–22.4 kHz. Significant results (P ≤ 0.05) are highlighted with an asterisk.

	**Band**	**Band**	**Band**	**Band**	**Band**	**Band**	**Band**
	**4.4–5.6 kHz**	**5.6–7.1 kHz**	**7.1–8.9 kHz**	**8.9–11.2 kHz**	**11.2–14.1 kHz**	**14.1–17.8 kHz**	**17.8–22.4 kHz**
**T value Short-beaked common dolphin**	0.97	1.39	1.74	3.11*	3.08*	4.44*	3.66*
**N = 26**							
**T value Atlantic Spotted dolphin**	0.77	1.44	1.58	3.03*	2.64*	4.06*	2.90*
**N = 84**							
**T value Striped dolphin**	1.14	1.46	1.41	2.04	1.98	2.34*	2.25*
**N = 16**							

### 2. Mean frequency values of whistles

The mean frequency values ranged from 7.2 kHz observed for the minimum frequency and 20.2 kHz for the maximum frequency of whistles emitted by short-beaked common dolphins, from 5.2 and 23.1 kHz for Atlantic spotted dolphin and from 7.1–20.4 kHz for striped dolphin (Table 4). We calculated the Coefficient of Variation for frequency parameters, which resulted the lowest for minimum frequency of whistles emitted by striped dolphins (CV = 9.22), but also for maximum frequency of Atlantic spotted and short-beaked common dolphins (respectively 10.32 and 10.09). Since the number of recordings considered is respectively of 16 for striped dolphin, 84 for Atlantic spotted dolphin and 26 for short-beaked common dolphin, the low CVs do not appear to be associated only with low sample sizes. The highest Coefficient of Variation was observed in the beginning frequency of Atlantic spotted dolphins (21.51).

**Table 4:** Mean, maximum, minimum, and standard deviation values of whistles frequency parameters (in kHz) considered in the study for short-beaked common, Atlantic spotted and striped dolphins.

		Beginning frequency	End frequency	Minimum frequency	Maximum frequency
Short-beaked common dolphin	Mean	**13.02**	**12.07**	**8.41**	**16.56**
	Maximum	18.14	16.51	10.54	20.21
	Minimum	8.28	8.71	7.24	12.83
	Sd	2.26	2.23	1.39	1.71
**N = 26**	Cv	**17.24**	**18.20**	**16.68**	**10.09**
Atlantic Spotted dolphin	Mean	**9.44**	**14.62**	**7.40**	**17.93**
	Maximum	19.88	22.76	12.55	23.13
	Minimum	6.17	7.87	5.24	10.62
	Sd	2.03	2.46	1.04	1.85
**N = 84**	Cv	**21.51**	**16.80**	**14.18**	**10.32**
Striped dolphin	Mean	**12.06**	**11.39**	**8.14**	**16.88**
	Maximum	15.93	13.33	10.33	20.47
	Minimum	8.83	8.16	7.11	11.18
	Sd	2.01	1.50	0.75	2.75
**N = 16**	Cv	**16.64**	**13.17**	**9.22**	**16.29**

**Fig 4:**
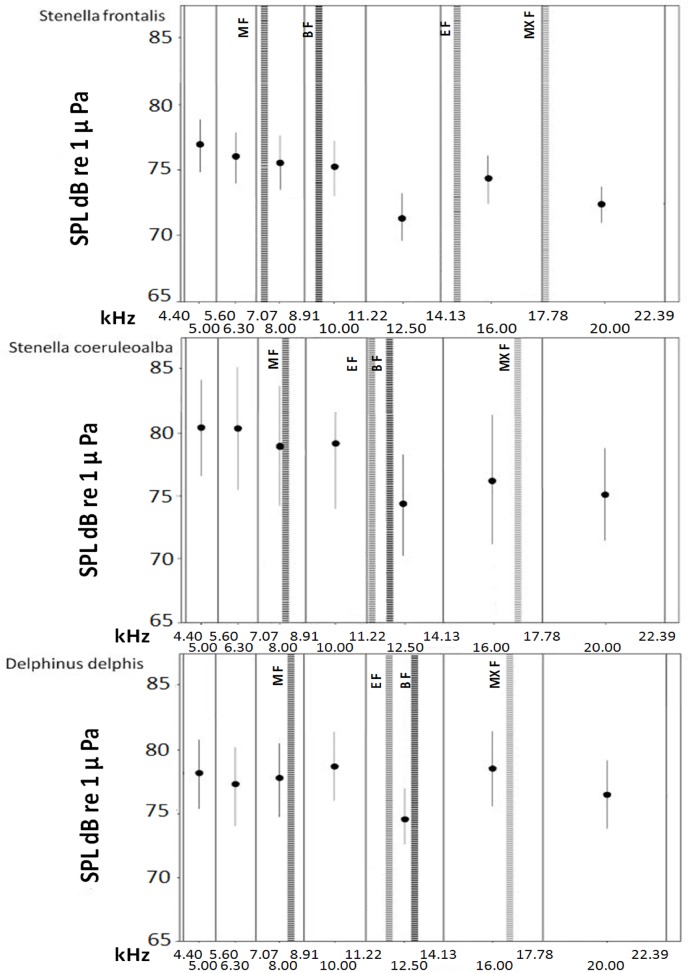
Distribution of the mean values of the frequency parameters considered in this study for *Stenella frontalis*, *Stenella coeruleoalba* and *Delphinus delphis* within the range of the noise one-third-octave bands. Circles represent the mean value and the standard error of SPLs of underwater noise, measured at the central frequency of each one-third-octave band for each species.

For all species, the mean minimum frequency is included within the band from 7.1 to 8.9 kHz the maximum frequency in the band from 14.1 to 17.8 kHz for striped and short-beaked common dolphins and in the band from 17.8 to 22.4 kHz for Atlantic spotted dolphins. Patterns of beginning and end frequency are more variable among species: the beginning frequency of the Atlantic spotted dolphin falls within the band from 8.9 to 11.2 kHz and the end frequency between the 14.1–17.8 kHz band, while the end frequency is lower than the beginning frequency and falls within the band from 11.2 to 14.1 kHz for striped and short-beaked common dolphins (Fig. 4).

### 3. Influence of noise levels on frequency parameters

We investigated variability of frequency parameters in relation to noise SPLs in each band. For striped dolphins, a positive correlation was observed between Δ frequency of minimum frequency parameters and values of SPLs in the bands from 4.4 to 5.6 kHz, as well as in bands from 5.6 to 7.1 kHz (Pearson’s correlation N = 16, Coefficient = 0.59, P = 0.02; Coefficient = 0.65, P = 0.01). For Atlantic spotted dolphins, positive correlations were observed between Δ frequency of end frequency parameter and values of SPLs in the bands from 14.1 to 17.8 kHz and from 17.8 to 22.4 kHz (Pearson’s correlation N = 84, Coefficient = 0.22, P = 0.04; Coefficient = 0.24, P = 0.03.) and between Δ frequency of maximum frequency parameter and the values of SPLs in the bands from 17.8 to 22.4 kHz (Coefficient = 0.23, P = 0.03). Since this species is the only one showing significant results for the end frequency, we checked separately for decibel variability. Given that a recent work on striped dolphins demonstrated a correlation between end frequency and the intensity of natural noise [19], we tested the hypothesis that the correlation is higher with SPLs in the range of noise from natural sources. We obtained the lowest p-value between Δ frequency of end frequency parameter and values of SPLs considering only SPLs in the 50 and 101 dB (band 14.1–17.8 kHz Coefficient = 0.29, P = 0.01; band 17.8–22.4 kHz Coefficient = 0.31, P = 0.01). Over 101 dB no significant correlations are observed.

For short-beaked common dolphins, a positive correlation was shown between Δ frequency of maximum frequency parameter and the values of SPLs in the bands 14.1–17.8 kHz, but only over the mean values in 2–3 weather condition (band 14.1–17.8 kHz Coefficient = 0.59, P = 0.01).

We tested for the predictability of parameters as a function of the SPLs values of all the bands from 4.4 and 22.4 kHz. Linear regression test confirmed that the variability of minimum frequency values observed for striped dolphin was strongly related to SPLs values (Linear Regression: R = 0.89, B = 6726.240, Sderr = 966.250, F = 4.5, P = 0.02), but not for other parameters and for none of the other two species.

Furthermore, we tested if SPLs recorded for each band and the effect of the interaction of SPLs recorded in the previous and/or subsequent bands, affected values of these whistle parameters, by mixed models (Table 5). The analysis confirmed that variability is attributable to SPLs for striped dolphin in minimum frequency and for the Atlantic spotted dolphins in end and maximum frequency. SPLs affected values of maximum frequency also for short-beaked common dolphins. Moreover, mixed models showed that the signal could be affected by interactions between SPLs of the noise band overlapping with the whistle mean frequency parameters and either previous or subsequent bands.

**Table 5:** Results of the GLMM: SPLs recorded for each band and the effect of the interaction of SPLs recorded in different bands, affected values of frequency parameters. The variability is attributable to SPLs for short-beaked common dolphins in maximum frequency, for Atlantic spotted dolphins in maximum, end, beginning and minimum frequency, and for striped dolphin in minimum and beginning frequency.

**Short-beaked common dolphin**					
	*Band*	*Estimates*	*Standard error*	*F*	*P*
**Maximum frequency**	11.2–14.1 kHz	113.629	75.357	2.274	0.145
**CV = 10.09**	14.1–17.8 kHz	325.389	71.796	20.540	**< 0.001**
	11.2–14.1 kHz*14.1–17.8 kHz	−2.867	0.492	33.935	**< 0.001**
**R** ^**2**^ = **0.070**					
**P = 0.433**					
	14.1–17.8 kHz	343.291	54.832	39.196	**< 0.001**
	17.8–22.4 kHz	77.169	63.551	1.474	0.237
	14.1–17.8 kHz *17.8–22.4 kHz	−2.611	0.461	32.081	**< 0.001**
**R** ^**2**^ = **0.149**					
**P = 0.1560**					
**Atlantic spotted dolphin**					
	*Band*	*Estimates*	*Standard error*	*F*	*P*
**Maximum frequency**	14.1–17.8 kHz	194.695	37.867	26.436	**< 0.001**
**CV = 10.32**	17.8–22.4 kHz	286.181	41.596	47.335	**< 0.001**
	14.1–17.8 kHz *17.8–22.4 kHz	−3.597	0.214	204.911	**< 0.001**
**R** ^**2**^ = **0.060**					
**P = 0.080**					
**End Frequency**	11.2–14.1 kHz	179.328	49.851	12.941	**< 0.001**
**CV = 16.80**	14.1–17.8 kHz	191.009	47.554	16.134	**< 0.001**
	11.2–14.1 kHz*14.1–17.8 kHz	−2.235	0.262	72.862	**< 0.001**
**R** ^**2**^ = **0.047**					
**P = 0.143**					
	14.1–17.8 kHz	153.002	45.843	11.139	**< 0.001**
	17.8–22.4 kHz	217.712	50.357	18.691	**< 0.001**
	14.1–17.8 kHz *17.8–22.4 kHz	−2.221	0.259	73.712	**< 0.001**
**R** ^**2**^ = **0.058**					
**P = 0.089**					
**Striped dolphin**					
	*Band*	*Estimates*	*Standard error*	*F*	*P*
**Minimum frequency**	5.6–7.1 kHz	143.100	24.865	33.121	**< 0.001**
**CV = 9.22**	7.1–8.9 kHz	37.975	24.029	2.498	0.139
	5.6–7.1 kHz*7.1–8.9 kHz	−0.958	0.107	80.375	**< 0.001**
**R** ^**2**^ = **0.733**					
**P < 0.001**					
	7.1–8.9 kHz	124.867	67.556	3.415	0.087
	8.9–11.2 kHz	56.288	72.728	0.598	0.453
	7.1–8.9 kHz*8.9–11.2 kHz	−0.939	0.127	54.488	**< 0.001**
**R** ^**2**^ = **0.204**					
**P = 0.227**					

## Discussion

The seminal works by Au et al. [33] supported later on by Lesage et al. [34] showed that in noisy habitats beluga whales alter their communication behaviour by shifting whistle frequencies. Among dolphins, only for *Tursiops ssp*. it was described that background noise could be the responsible for shifting frequency towards higher values [16, 18, 17], although this was hypothesised also for short-beaked common dolphins [35]. Nevertheless, all these studies refer to anthropogenic noise produced by boat engines.

In the present study, we compared the effects of natural and anthropogenic noises on the acoustic communication of three species of dolphins and we found that they use a common strategy: dolphins modified the acoustic structure of whistles in association with increasing levels of the environmental background noise occurring within the frequency bands of social communication. Furthermore, our results show that noise selectively influences various parameters of the acoustic signal. Dolphins appear to adopt a noise-induced vocal compensation, thereby giving rise to species-specific shifts in frequency parameters, and finally increasing the frequency of their calls according to the level of environmental background noise. This is probably a compensatory strategy to maintain threshold levels favourable for communication.

Atlantic spotted dolphins and short-beaked common dolphins increased the maximum frequency of their whistles in presence of high noise levels. This is in agreement with previous research on the bottlenose dolphins occurring in the Atlantic Ocean and in the Mediterranean Sea [16, 18]. The striped dolphin apparently used a different strategy: it strongly increased the minimum frequency, which shows limited variability. This is the only species where an acoustic feature linearly changed with increasing noise levels.

Maximum and minimum frequency have been recognised as the parameters having the lowest within species variability (maximum frequency for short-beaked common [35, 36, 37, 25] and Atlantic spotted dolphins [20, 38, 39, 40]; minimum frequency for the striped dolphins [19, 41, 24]). Minimum and maximum frequency, in fact, are known for being linked to morphological constraints [21, 20, 22, 16, 19, 24,25]. Steiner [42] and Wang et al. [20] suggested that these variables could represent species-specific features of whistles, because of their stability within certain ranges, and can provide conspecific listeners with species-specific cues. Therefore, dolphins modify their most stable acoustic parameter, a trait related to their species-specific features. Nevertheless, each species shows a different potential of modifying the acoustic structure of this signal. It would be interesting to perform further studies on the consequences of these changes, i.e. to assess the potential costs of altering key species-specific parameters.

No alteration of the most stable parameters may be required to make communication possible in the presence of moderated increases in environmental noise. From our results, we can hypothesize that adjustments of the end frequency may be useful to overcome fluctuating noise levels originating from natural sources. For the Atlantic spotted dolphin the Δ frequency of end frequency parameter correlated with SPLs especially when we used for the analysis only levels included in the range of the noise originating from natural sources. This pattern may indicate a strategy to increase end frequency only in relation to a moderate increase in environmental noise. In other species, such as in non-human primates [43] and birds [44], the end of the call is the part having lowest amplitude and is the most strongly influenced by degradation, absorption and reverberation over distance. Since our findings complements results by Azzolin et al. [19], who showed that the end frequency of Mediterranean striped dolphins is positively correlated with wind intensity (the main environmental component of mid-frequency noise), we can hypothesize that, also in dolphins, the increase in end frequency may be a modality for improving transmission efficiency.

Furthermore, we found significant interactions with environmental noise within a range of frequency and not only with the SPL in the frequency band overlapping with the mean value of the whistle parameters considered. In this study, recorded noise ranged from 19 to 120 dB re 1 μ Pa and, when conditions deteriorated (2–3 or over standard weather conditions), it increased especially from 4.4 to 11.2 kHz. We found that ambient noise level and synergy among SPLs in the different bands (as highlighted by results of the linear mixed models) have a role in shaping whistles' structure, thereby providing further evidence that dolphins' acoustic signals are influenced by multiple pressures originating from the environment [45, 15, 16, 19].

We found that the masking compensation process results from a balance between noise intensity and constraints imposed to the features of whistles. Compensation works in the framework of the general rules proposed by Endler [46], suggesting that dolphins should:

1. Choose frequency bands which minimize background noise.

In our case, the frequency shifts, together with the low variability of the involved parameters may be a sender's crucial modality to avoid masking by environmental background noise and to facilitate an efficient communication by the transmission of meaningful information.

1. Use species-specific frequency bands and tuned receptors in order to minimize noise at other frequencies.

Calls should occupy a frequency band where conspecifics have high auditory sensitivity. A stronger increase or decrease in these parameters could be inefficient for both the sender and the receiver. Dolphin signals are not only adaptive [15], but can be actively modified within the species-specific range, to cope with noisy environments. Nevertheless, the fine-scale biological significance of whistles’ acoustic variability and the costs of compensation remain unclear, especially in areas where intense noise often occurs

## References

[1] Richardson WJ, Greene CR, Malme CI, Thomson DH (1995) Marine mammals and noise Academic Press, San Diego, CA. 576 p

[2] Bateson M (2007) Environmental noise and decision making, possible implications of increases in anthropogenic noise for information processing in marine mammals. Int J Comp Psychol 20: 169–178.

[3] Parks SE, Johnson M, Nowacek D, Tyack PL (2010) Individual right whales call louder in increased environmental noise. Biol Letters. 10.1098/rsbl.2010.0451 PMC3030867 20610418

[4] Ketten DR (1992) The marine mammal ear: specializations for aquatic audition and echolocation In: The Evolutionary Biology of Hearing, Webster D. B., Fay R.R., and Popper A. N. (eds.), Springer-Verlag, pp. 717–750.

[5] Nowacek DP, Thorne LH, Johnston DW, Tyack PL (2007) Responses of cetaceans to anthropogenic noise. Mammal Rev 37: 81–115.

[6] Dolman SJ, Simmonds MP (2006) An updated note on the vulnerability of cetaceans to acoustic disturbance. SC/58/E22 Scientific Committee of the International Whaling Commission

[7] Miksis-Olds JL, Tyack PL (2009) Manatee (*Trichechus manatus*) vocalization usage in relation to environmental noise levels. J Acoust Soc Am 125: 1806–1815. 10.1121/1.3068455 19275337

[8] Ellison WT, Southall BL, Clark CV, Frankel AS (2011) A new context-based approach to assess marine mammal behavioral responses to anthropogenic sounds. Conserv Biol. 10.1111/j.1523-1739.2011.01803.x 22182143

[9] Clark CW, Ellison WT, Southall BL, Hatch L, Van Parijs SM, Frankel A, et al (2009) Acoustic masking in marine ecosystems: intuitions, analysis, and implication. Mar Ecol Prog Ser 395: 201–222.

[10] Gelfand SA (2004) Hearing—an introduction to psychological and physiological acoustics Marcel Dekker, New York.

[11] Hatch LT, Clark CW, Van Parijs SM, Frankel AS, Ponirakis DW (2012) Quantifying loss of acoustic communication space for right whales in and around a US National Sanctuary. Conserv Biol 26: 983–994 10.1111/j.1523-1739.2012.01908.x 22891747

[12] Branstetter BK, Trickey JS, Bakhtiari K, Black A, Aihara H, Finneran JJ (2013) Auditory masking patterns in bottlenose dolphins (*Tursiops truncatus*) with natural, anthropogenic, and synthesized noise J Acoust Soc Am 133: 1811–1818. 10.1121/1.4789939 23464049

[13] Holt MM, Noren DP, Veirs V, Emmons CK, Veirs S (2009) Speaking up: Killer whales (*Orcinus orca*) increase their call amplitude in response to vessel noise. J Acoust Soc Am 125: EL27–EL32. 10.1121/1.3040028 19173379

[14] Jensen FH, Bejder L, Wahlberg M, Aguilar Soto N, Johnson M, Madsen PT (2009) Vessel noise effects on delphinid communication. Mar Ecol Prog Ser 395: 161–175.

[15] Morisaka T, Shinohara M, Nakahara F, Akamatsu T (2005a) Effects of ambient noise on the whistles of Indo-Pacific bottlenose dolphin populations. J Mammal, 86: 541–546.

[16] May-Collado LJ, Wartzok D (2008) A comparison of bottlenose dolphin whistles in the Atlantic Ocean: factors promoting whistle variation. J Mammal 89: 1229–1240.

[17] Luis AR, Couchinho MN, Dos Santos ME (2014) Changes in the acoustic behavior of resident bottlenose dolphins near operating vessels. Mar Mam Sci. 10.1111/mms.12125

[18] La Manna G, Manghi M, Pavan G, Lo Mascolo F, Sarà DG (2013) Behavioural strategy of common bottlenose dolphins (Tursiops truncatus) in response to different kinds of boats in the waters of Lampedusa Island (Italy). Aquat Cons Mar Fresh Eco 23: 745–757.

[19] Azzolin M, Papale E, Lammers MO, Gannier A, Giacoma C (2013) Geographic variation of whistles of striped dolphin (Stenella coeruleoalba) within the Mediterranean Sea. J Acoust Soc Am 134: 694–705 10.1121/1.4808329 23862842

[20] Wang D, Würsig B, Evans WE (1995) Comparisons of whistles among seven odontocete species In Kastelein RA, Thomas JA, and Nachtigall PE. Sensory systems of aquatic mammals De Spil Publishers, Woerden, Netherlands Pp. 299–323

[21] Rendell LE, Matthews JN, Gill A, Gordon JCD, Macdonald DW (1999) Quantitative analysis of tonal calls from five odontocete species, examining interspecific and intraspecific variation. J Zool (London), 249:403–410.

[22] Oswald JN, Barlow J, Norris TF (2003) Acoustic identification of nine delphinid species in the eastern tropical pacific ocean. Mar Mam Sci 19: 20–37.

[23] Morisaka T, Shinohara M, Nakahara F, Akamatsu T (2005b) Geographic variations in the whistles among three Indo-Pacific bottlenose dolphin *Tursiops aduncus* populations in Japan. Fish Sci 568–576.

[24] Papale E, Azzolin M, Cascão I, Gannier A, Lammers MO, Martin VM, et al (2013a) Geographic variability in the acoustic parameters of striped dolphin’s (Stenella coeruleoalba) whistles. J Acoust Soc Am 133: 1126–1134. 10.1121/1.4774274 23363128

[25] Papale E, Azzolin M, Cascão I, Gannier A, Lammers MO, Martin VM, et al. (2013b) Macro and micro geographic variation of short-beaked common dolphin’s (*Delphinus delphis*) whistles in the Mediterranean Sea and Atlantic Ocean. Ethol Ecol Evol.

[26] Evans PGH, Hammond PS (2004) Monitoring cetaceans in European waters. Mammal Rev 34: 131–156.

[27] Connor RC, Smolker RA, Richards AF (1992) Two levels of alliance formation among male bottlenose dolphins (*Tursiops sp*). Proc Natl Acad Sci 89: 987–990. 11607275 10.1073/pnas.89.3.987 PMC48370

[28] Tyack PL (1997) Development and social functions of signature whistles in bottlenose dolphins, *Tursiops truncatus* . Bioacoustics 8: 21–46.

[29] Smolker R, Pepper JW (1999) Whistle convergence among allied male bottlenose dolphins (delphinidae, Tursiops sp). Ethology, 105: 595–618.

[30] Boersma P, Weenink D (2013) Praat: doing phonetics by computer. Version 5.3.56. Available: http://www.praat.org/. Accessed: 2013 Sep 15.

[31] Gamba M, Colombo C, Giacoma C (2012) Acoustic cues to caller identity in lemurs: a case study. J Ethol. 10.1007/s10164-011-0291-z

[32] Erbe C (2002) Underwater noise of whale-watching boats and potential effects on killer whales (Orcinus orca), based on an acoustic impact model. Mar Mam Sci 18: 394–418.

[33] Au WWL, Carder DA, Penner RH, Scronce B (1985) Demonstration of adaptation in Beluga whale echolocation signals. J Acoust Soc Am 772:726–730. 10.1121/1.392341 3973242

[34] Lesage V, Barrettme C, Kingsleaynd CS, Sjare B (1999) The effect of vessel noise on the vocal behavior of belugas in the St. Lawrence River estuary, Canada. Mar Mam Sci 15: 65–84.

[35] Ansmann IC, Goold JC, Evans PGH, Simmonds M, Keith SG (2007) Variation in the whistle characteristics of short-beaked common dolphins, *Delphinus delphis*, at two locations around the British Isles. J Mar Biol Assoc UK 87: 19–26.

[36] Oswald JN, Rankin S, Barlow J, Lammers MO (2007) A tool for real-time acoustic species identification of delphinid whistles. J Acoust Soc Am 122: 587–595. 17614515 10.1121/1.2743157

[37] Petrella V (2009) Whistle characteristics of common dolphins (*Delphinus sp*.) in the Hauraki Gulf, New Zealand. PhD dissertation in Applied Biology, University of Naples, Italy.

[38] Lammers MO, Au WWL, Herzing DL (2003) The broadband social acoustic signaling behavior of spinner and spotted dolphins. J Acoust Soc Am 114, 1629–1639. 14514216 10.1121/1.1596173

[39] Baron SC, Marinez A, Garrison LP, Keith EO (2008) Differences in acoustic signals from delphinids in the western North Atlantic and northern Gulf of Mexico. Mar Mam Sci 24: 42–56.

[40] Azevedo AF, Flach L, Bisi TL, Andrade LG, Dorneles PR, Lailson-Brito J (2010) Whistles emitted by Atlantic spotted dolphins (Stenella frontalis) in southeastern Brazil. J Acoust Soc Am 127: 2646–2651 10.1121/1.3308469 20370045

[41] Azzolin M, Gannier A, Lammers MO, Oswald JN, Papale E, Buscaino G, et al (2014) Combining whistles acoustic parameters to discriminate Mediterranean odontocetes for their Passive Acoustic Monitoring. J Acoust Soc Am 135: 502–512. 10.1121/1.4845275 24437790

[42] Steiner WW (1981) Species-specific differences in pure tonal whistle vocalizations of five western North Atlantic dolphin species. Behav Ecol Sociobiol, 9: 241–246.

[43] Maciej P, Fischer J, Hammerschmidt K (2011) Transmission Characteristics of Primate Vocalizations: Implications for Acoustic Analyses. PLoS ONE 6(8): e23015 10.1371/journal.pone.0023015 21829682 PMC3148239

[44] Slabbekoorn H, Ellers J, Smith TB (2002) Birdsong and sound transmission: the benefits of reverberations. The Condor 104: 564–573.

[45] Janik VM, Slater PJB (2000) The different roles of social learning in vocal communication Anim Behav 60: 1–11. 10924198 10.1006/anbe.2000.1410

[46] Endler JA (1992) Signals, signal conditions, and the direction of evolution. The American Naturalist 139: S125–S153.

